# 

**Published:** 1811-01

**Authors:** 


### THE MEDICAL AND PHYSICAL JOURNAL.

CONDUCTED BY

SAMUEL FOTHERGILL, M.D.

AND

WILLIAM ROYSTON, ESQ.

VOL. XXV.

FROM JANUARY TO JUNE, 1811.

Et quoniam variant morbi, variabimus artes;
Mille mali species, mille salutis erunt.

5/31

*LONDON:*

PRINTED FOR RICHARD PHILLIPS, NO. 7, BRIDGE-STREET, BLACKFRIARS.

BY E. HEMSTED, GREAT NEW STREET, FETTER LANE.

### MONTHLY CATALOGUE OF NEW MEDICAL PUBLICATIONS.

On Diseases of the Generative System; with twelve plates. By John Roberton. 8vo. Highley.

The Anatomy of the Human Bones and Nerves. By Alexander Monro, M. D. Highley.

Observations on the Natural History, Climate, and Diseases of Madeira, during a period of eighteen years. By William Gourlay, M. D. Callow.

Practical Observations on the Sclerocele, and other Morbid Enlargements of the Testicle, also on the cause and cure of the Acute, the Spurious, and the Chronic Hydrocele. The whole illustrated by cases, to which are added four cases of operations for aneurism, subclavian, femoral, popliteal, and femoral-popliteal, with Practical Remarks, and Plates. By Thomas Ramsden. 8vo. Wilkie and Robinson.

Memoirs of the Life of Thomas Beddoes, M. D. with an Analytical account of his Writings. By John Edmonds Stock, M. D. 4to. Murray.

A System of Chemical Philosophy. Part 2d. By John Dalton, 8vo. Bickerstaff.

Dissertation on Insanity; Illustrated with tables, and extracted from between two and three thousand cases in Bedlam. By William Black, M. D. 8vo.

### NOTICES TO CORRESPONDENTS.

From the account of the Society for the Relief of the Widows and Orphans of Medical men, in the last number of our Journal, Mr. Chamberlain will find that the plan of that philanthropic institution, with which he has favoured us, need not now be inserted.

We have been obliged to defer till our next number, the conclusion of the interesting paper of Medicus upon Tobacco; also amongst other articles, communications from H. R. from Dr. Hamilton of Ipswich, Dr. Cookson, Dr. Merriman, and Mr. Bailey, &c. &c.

*The following notices came to hand too late to be inserted in their proper places.*

Mr. Stevenson, Great Russell-street, Bloomsbury, (author of "a Practical Treatise on the *Morbid Sensibility* of the *Eye*, commonly called *Weakness* of *Sight*,") purposes delivering a Course of Lectures on the Anatomy, Physiology, and Diseases of the *Eye* and *Ear*, early in the ensuing spring.

Dr. Reid will commence his Spring course of Lectures on the Theory and Practice of Medicine, on Wednesday the 24th of January.

Joseph Reade, M. D. of Cork, has in the press, a treatise entitled, Critical and Practical Observations on the Diseases of the inner corner of the Human Eyes, comprising the epiphora, the tumor sacculi lachrymalis, and the fistula lachrymalis, with a new arrangement and method of Cure.

Mr. Parkinson is about to publish Observations on the Act for Regulating Mad-houses, with remarks addressed to the friends of the Insane; and a correction of the mistatements of the case of Benjamin Elliot, sentenced to six Months imprisonment for illegally depriving Mary Daintree of her liberty.

Surgical Observations on Injuries of the Head, and on Miscellaneous Subjects. By John Abernethy. F. R. S. 8vo. boards 7s. Longman and Co.

*Printed by E. Hemsted, Great New Street, Fetter Lane.*

